# Conflicts and Collisions With an Endangered Carnivore: Landscape Drivers and Spatial Risk Pattern

**DOI:** 10.1002/ece3.73931

**Published:** 2026-07-03

**Authors:** Marta Prat‐Guitart, David P. Onorato, James E. Hines, Madan K. Oli

**Affiliations:** ^1^ Department of Wildlife Ecology and Conservation University of Florida Gainesville Florida USA; ^2^ Florida Fish and Wildlife Conservation Commission Fish and Wildlife Research Institute Naples Florida USA; ^3^ Eastern Ecological Sciences Center U.S. Geological Survey Laurel Maryland USA

**Keywords:** carnivore conservation, depredation, human–wildlife conflict, landscape ecology, occupancy modeling, vehicular collision

## Abstract

Human–wildlife conflicts and anthropogenic mortality are expected to increase with the continuous encroachment on natural habitats within and around protected areas, yet the processes governing these risks remain poorly understood. We investigated the spatiotemporal dynamics and drivers of two distinct processes involving the endangered Florida panther ( *Puma concolor coryi* ; hereafter, panther): (1) direct human–panther interactions and livestock depredation (hereafter, conflicts), and (2) vehicle collisions (hereafter, collisions). Using long‐term data collected between 2006 and 2022 within the panther's breeding range in Southwest Florida, USA, we applied dynamic occupancy models to estimate probabilities of occurrence, colonization, and extinction of conflicts and collisions while accounting for imperfect detection. A total of 277 conflicts and 239 collisions were recorded during the study period. Occurrence probabilities for both conflicts and collisions increased during the early years of the study and subsequently stabilized. Landscape configuration consistently showed strong associations with both processes. Conflicts were more likely in areas with fragmented but closely spaced habitat patches, whereas collisions were more likely along road segments characterized by connected landscapes with low resistance to panther movement. In contrast, areas that showed greater distances between habitat patches and increased proportions of protected area were associated with lower probabilities of conflict or collision persistence over time. A predictive map was created to highlight areas with high probability of conflict and road segments with high probability of collision in Southwest Florida, USA. Identifying areas with elevated risk can inform targeted mitigation strategies, including habitat management, wildlife crossings, and outreach efforts, and further support long‐term coexistence between humans and large carnivore populations.

## Graphical abstract

Using long‐term data (2006–2022) and dynamic occupancy models, we investigated the spatiotemporal dynamics and landscape drivers of human–wildlife conflicts and vehicle collisions involving the endangered Florida panther in Southwest Florida. Both processes were consistently associated with landscape configuration, yet through different mechanisms: conflicts were more likely in fragmented but closely spaced habitat patches, while collisions were more likely along roads traversing well‐connected landscapes with low resistance to panther movement. Identifying areas with elevated risk can inform targeted mitigation strategies and further support long‐term coexistence between humans and large carnivore populations.

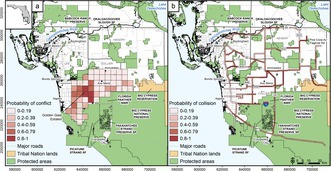

## 1 Introduction

Large wildlife species require extensive space to meet their energetic and reproductive needs, resulting in frequent overlaps between their activities and human‐dominated landscapes. As human development expands and natural habitats become increasingly fragmented, this spatial overlap intensifies, leading to a range of conflicts between humans and wildlife (Loveridge et al. 2010; Wittemyer et al. 2008; Woodroffe et al. 2005). When the species in question is a large carnivore, these conflicts can result in negative outcomes for both humans and wildlife, including livestock depredation, threats to human safety, and increased mortality risk for carnivores, with consequences for livelihoods, public attitudes, and long‐term population persistence (Inskip and Zimmermann 2009; Loveridge et al. 2010; Oli et al. 1994; Puri et al. 2022; Ripple et al. 2014; Thirgood et al. 2005; Treves and Karanth 2003; Woodroffe et al. 2005).

In addition to conflicts, wildlife and humans interact through vehicle collisions (hereafter, collisions), which can represent a significant source of human‐related mortality for large carnivores (van de Kerk et al. 2019). Because of their extensive range requirements, these species frequently traverse heterogeneous landscapes, including roads and other linear infrastructure that increase the likelihood of mortality through collisions (Loveridge et al. 2010; Schwab and Zandbergen 2011; Swanson et al. 2008). Roads can also impede movement, fragment populations, and disrupt demographic and genetic connectivity (Dixon et al. 2006; Jackson and Fahrig 2011), effectively transforming otherwise suitable habitats into population sinks (Woodroffe and Ginsberg 1998).

Understanding and managing the drivers and dynamics of human–carnivore conflicts and collisions is therefore critical for their conservation and coexistence with humans (Dickman 2010; Inskip and Zimmermann 2009). However, these processes are often examined separately and are rarely studied within a unified spatiotemporal framework that accounts for imperfect detection and changing population dynamics (but see Goswami et al. 2015; Moore et al. 2018; Puri et al. 2022; Santos et al. 2022). Imperfect detection can arise when events go unobserved or unreported, such as an unconfirmed depredation or a collision that is not reported to management agencies (McClintock et al. 2015). Hence, ignoring imperfect detection can bias inference about occurrence and temporal dynamics (MacKenzie et al. 2017, 2002). This is particularly relevant for human–carnivore conflicts and collisions, where reporting likely varies across space and land‐use contexts. Dynamic occupancy models provide a useful framework for quantifying these processes by estimating probabilities of occurrence, emergence (colonization), and cessation (extinction) across space and time.

Recovering carnivore populations provide a valuable context for investigating these dynamics. As populations increase and expand into human‐dominated landscapes, conflict with humans and exposure to anthropogenic risks are expected to change. In this context, the Florida panther ( *Puma concolor coryi* ; hereafter, panther) provides an ideal system for examining how conflict and collision risk arise during population recovery. Once reduced to an estimated 20–30 individuals in the 1990s (McBride et al. 2008), the population has increased to approximately 200 individuals following conservation measures that included habitat protection, genetic rescue, and mitigation of road mortality (Foster and Humphrey 1995; Hostetler et al. 2010; Johnson et al. 2010; Lotz et al. 1997; Onorato et al. 2010, 2024; van de Kerk et al. 2019). Despite this recovery, the population remains restricted to less than 5% of its historic range within a landscape characterized by rapid human development and extensive road networks (Davis et al. 2021; Onorato et al. 2010). As a result, both human–panther conflicts and collisions have increased and represent ongoing challenges for conservation and management (Benson et al. 2011; Kautz et al. 2006; Onorato et al. 2010; Rodgers and Pienaar 2017, 2018; van de Kerk et al. 2019). Previous studies have examined aspects of these processes, including livestock depredation (Jacobs et al. 2015), public perception (Langin and Jacobson 2012; Rodgers and Pienaar 2017), and collisions (Schwab and Zandbergen 2011), but a comprehensive assessment of their spatiotemporal dynamics and shared or distinct drivers remains incomplete.

In this study, we aimed to: (1) quantify the spatiotemporal dynamics of human–panther direct interactions and depredations (hereafter, conflicts) and collisions; (2) identify ecological, climatic, and anthropogenic drivers influencing these processes; and (3) develop spatially explicit predictions of conflict and collision risk to inform management strategies in Southwest Florida, USA. To account for imperfect detection, we applied a dynamic occupancy modeling framework (MacKenzie et al. 2017) to long‐term data on conflicts and collisions. We hypothesized that, as the panther population expanded and the overlap with human‐dominated landscapes increased, the probabilities of conflict and collisions would increase initially due to expanding overlap with human activities, but that these dynamics would also be mediated in part by ecological drivers that influence panther resources and movement.

## 2 Materials and Methods

### 2.1 Study Area

**FIGURE 1:**
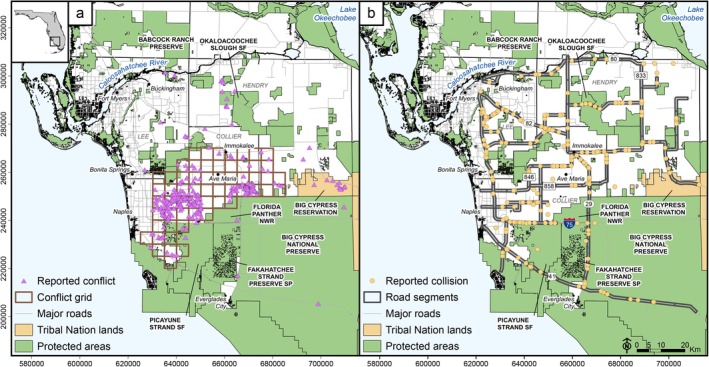
Study area and locations of human‐panther conflicts and vehicle collisions in Southwest Florida, USA, between October 2006 and July 2022: (a) conflicts (purple triangles) and conflict grid (brown grid cells); and (b) collisions (yellow points) and collision road segments (black rectangles). NWR, National Wildlife Refuge; SF, State Forest; SP, State Park.

The study area encompassed public and private lands south of the Caloosahatchee River through Collier, Hendry, and Lee Counties, Florida, USA (Figure 1; Kautz et al. 2006; Onorato et al. 2011). This region supports the only breeding population of Florida panthers and represents one of the fastest‐growing human–wildlife interfaces in the southeastern United States. Major land uses include pastures, agricultural lands, residential and industrial areas, transportation infrastructure, and natural habitats such as hardwood hammocks, cypress forests, pine flatwoods, freshwater marshes, prairies, and grasslands (Kautz et al. 2006; Land et al. 2008; Onorato et al. 2011). The climate is subtropical with distinct wet (May–October) and dry (November–April) seasons (Criffield et al. 2018).

### 2.2 Human–Panther Conflicts

Conflict data were collected, verified, and georeferenced by the Florida Panther Interagency Response Team. Interactions were classified into five categories based on panther behavior, attractants to the area, and human activity (USFWS 2008): sighting (visual observation or fleeting glimpse of a panther from a distance); incident (encounter with a panther displaying potentially threatening behavior); threat (the panther presents unprovoked aggressive/predatory behavior toward a human that requires the individual to take defensive action to avoid direct contact); attack (direct, physical contact between a panther and a human involving aggressive panther behavior); and indirect interactions such as depredation on domestic pets or livestock. In this study, we included conflicts representing potential threats to humans (encounters, incidents, threats, and attacks) and depredation of domestic animals between October 2006 and July 2022.

For the conflict analysis, we defined a focal sampling domain primarily within Collier County representing landscapes where panther presence, human activity, and conflict reporting overlap (Figure 1a). This domain encompasses a mosaic of protected areas, rural lands, exurban development, and suburban interfaces where conflicts are ecologically plausible and consistently detectable. Areas outside this domain, particularly intensively urbanized regions and remote areas with reduced human–panther overlap, were not included because conflict processes are either structurally rare or subject to heterogeneous detection probabilities. Including such areas could conflate true absence with nondetection and violate key assumptions of occupancy models regarding detection and state processes. Accordingly, inference from the conflict models is conditional on landscapes with nonzero exposure to conflict risk, rather than representing the entire panther breeding range. This approach allows estimation of covariate effects on conflict dynamics within ecologically relevant and consistently monitored landscapes while avoiding bias introduced by large areas of structural zeros and inconsistent reporting.

The study grid covered 1475 km^2^ and was divided into 59 grid cells of 25 km^2^. Grid cell size was selected based on average daily panther movement distances (4.8 km; Criffield et al. 2018) to represent ecologically meaningful spatial units for occupancy analysis (MacKenzie et al. 2017).

### 2.3 Vehicle Collisions

Data on panther mortality and injury were obtained from the Florida Fish and Wildlife Conservation Commission (FWC), which maintains a long‐term database of verified cases (FWC 2025). In this study we included collisions occurring between October 2006 and July 2022 within the panther breeding range south of the Caloosahatchee River (Figure 1b). Because collisions are spatially constrained to road networks, we defined sampling units as road segments rather than grid cells. Major roads were divided into segments of approximately 10 km, covering a total road length of 804.3 km. When terminal segments were shorter than 10 km, segments ≥ 5 km were retained as independent units, whereas shorter segments were merged with adjacent segments to ensure sufficient spatial support for inference.

To characterize the landscape context influencing collision risk, we applied a 0.5 km buffer on each side of each road segment. At road intersections, major roads were maintained as continuous segments, whereas intersecting roads were split, beginning immediately after the buffered intersection area. This buffer represents the immediate road‐adjacent zone within which panthers approach, cross, or move along roads. While broader landscape structure influences panther movement at larger scales, collision risk is ultimately determined by fine‐scale interactions between animal movement and road infrastructure at crossing locations. Accordingly, covariates were summarized within this buffer to capture conditions directly associated with collision occurrence.

Road segments containing continuous wildlife crossing structures and fencing along Interstate 75 were excluded because these mitigation measures substantially reduce collision risk relative to other roads (Rytwinski et al. 2016), and their inclusion would violate the assumption of comparable exposure across sampling units.

### 2.4 Covariates

We extracted ecological, climatic, and anthropogenic covariates (Table 1) for both conflict and collision analyses using spatial overlays in the *terra* R package (Hijmans et al. 2024). Covariates were selected to represent key processes hypothesized to influence conflicts and collision risk, including habitat availability, landscape configuration, landscape resistance, and human activity (Table S1). For the conflict analysis, covariates were summarized at the grid‐cell level, reflecting landscape conditions influencing the probability of encounters between panthers and humans or their domestic animals. For the collision analysis, covariates were calculated within buffers surrounding road segments, representing the local landscape context at sites where panthers approach and cross roads.

**TABLE 1:** Description of site‐ and season‐level covariates used to assess drivers of human–panther conflicts and vehicle collisions in Southwest Florida, USA. Covariates were included in both analyses unless otherwise noted.

Covariate	Description
Panther population estimate	Number of panthers based on population estimates reported in Onorato et al. (2024) for 2006–2020.
Proportion of panther habitat	Land cover classes (FWC 2016) were classified as favorable (e.g., upland forest, wetland forest, marsh–shrub–swamp, prairie grassland) or unfavorable (e.g., agricultural, urban, open water, mangrove, exotic vegetation) based on panther movement and habitat selection patterns from Onorato et al. (2011). The proportion of favorable habitat was calculated for each sampling unit. Reclassification table included in Table S2.
Proportion of protected area	Proportion of protected land within each sampling unit based on the Florida Managed Lands Layer (FNAI 2016).
Landscape resistance (resistance score)	Land cover classes (FWC 2016) were reclassified to represent resistance to panther movement (1–20 scale; lower values indicate higher suitability for movement), following Kautz et al. (2006). Mean resistance was calculated for each sampling unit. Reclassification table included in Table S2.
Least‐cost distance to protected area^a^ (km)	Least‐cost distance from each grid cell to the nearest protected area (> 1 km^2^), calculated using the *gdistance* R package (van Etten 2017).
Patch aggregation (index)	Patch cohesion index describing the connectedness of favorable habitat patches within each sampling unit, calculated using the *landscapemetrics* R package (Hesselbarth et al. 2019).
Distance between patches (m)	Mean Euclidean nearest‐neighbor distance between favorable habitat patches within each sampling unit, calculated using the *landscapemetrics* R package (Hesselbarth et al. 2019).
Maximum temperature (°C)	Mean monthly maximum temperature derived from climate station data (NOAA 2024) and averaged for each sampling unit.
Total precipitation (mm)	Total monthly precipitation derived from climate station data (NOAA 2024) and averaged for each sampling unit.
Human density (people km^−2^)	Estimated human density per sampling unit based on U.S. Census data (U.S. Census Bureau 2024).
Livestock heads^a^	Number of cattle ( *Bos taurus* ) within Collier County (USDA NASS 2024).
Proportion of fencing around wildlife crossings^b^	Proportion of road length covered with fencing associated with wildlife crossings for each road segment (FDOT 2024a).
Road width^b^ (m)	Mean road width for each road segment (FDOT 2024b).
Road shoulder width^b^ (m)	Mean shoulder width for each road segment (FDOT 2024b).
Maximum speed limit^b^ (km h^−1^)	Maximum speed limit for each road segment (FDOT 2024; supplemented with Google Maps where necessary).
Traffic volume^b^ (vehicles day^−1^ km^−1^)	Annual average daily traffic per kilometer for each road segment (FDOT 2024b).
Grid area^b^ (m^2^)	Area of each road segment, included to account for variation in road segment length.

^a^ Covariate included only in conflict models.

^b^ Covariate included only in collision models.

**FIGURE 2:**
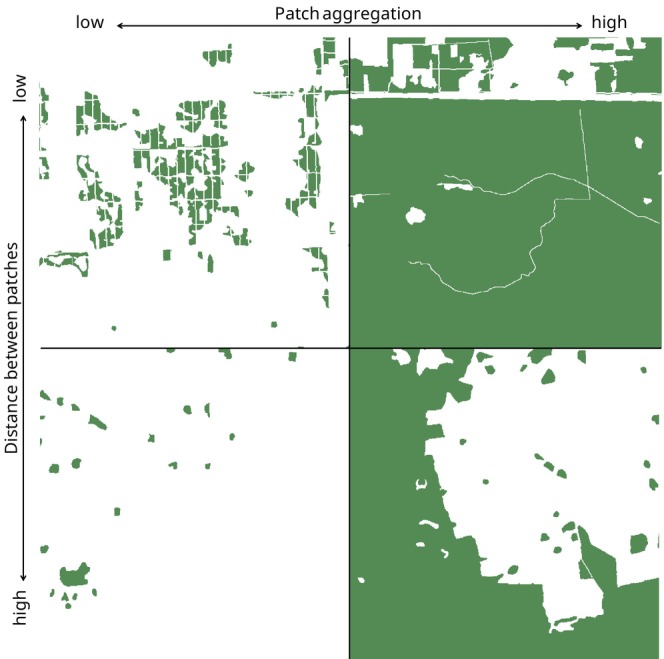
Graphic representation of landscape metrics used to measure landscape configuration: Patch aggregation and distance between habitat patches. Further descriptions of these covariates are provided in Table 1.

Panther habitat covariates were derived from land cover data (FWC 2016), with land‐cover classes categorized as favorable or unfavorable based on published habitat selection patterns. These covariates quantified both the proportion of suitable habitat and its spatial configuration within each grid cell, including metrics of patch aggregation and distance between habitat patches (Figure 2). Landscape resistance was derived from the same land cover data, reclassified to represent resistance to panther movement. Anthropogenic covariates described human land use and infrastructure associated with potential conflict or collision risk, including human population density, livestock abundance, and road characteristics such as traffic volume, speed limit, road and shoulder width, and fencing along wildlife crossings. Climate covariates included monthly maximum temperature and total precipitation. Detailed covariate descriptions are included in Table 1.

Covariates were calculated by sampling unit (grid cell or road segment) and season when temporal data were available and treated as constant otherwise. Missing values were replaced with mean values for the corresponding covariate. Continuous covariates were standardized (mean = 0, SD = 1) to facilitate model convergence and comparison of effect sizes. Correlations among covariates were evaluated prior to analysis. Highly correlated variables (|*r*| ≥ 0.7) included the proportion of panther habitat, landscape resistance, and human population density. These covariates were allowed in separate model parameters but were not included together within the same parameter.

### 2.5 Dynamic Occupancy Modeling

We used multi‐season occupancy models (MacKenzie et al. 2017, 2003) to evaluate the occurrence and temporal dynamics of conflicts and collisions. In this framework, “occupancy” represents the probability that at least one conflict or collision occurred within a sampling unit (grid cell or road segment, respectively) during a primary sampling season and therefore reflects the propensity for events to occur rather than continuous presence. Primary sampling seasons (*i*) extended from October 1 to July 31 of the following year, during which closure was assumed, with each month within this period considered as a secondary sampling occasion (*j*). Because August and September contained the fewest reported events, they were excluded to provide the recommended transition period between primary sampling seasons.

Closure within primary sampling seasons implies that the underlying state, whether a sampling unit is susceptible to conflicts or collisions, is assumed constant within a primary sampling season, while allowing changes between primary seasons (MacKenzie et al. 2017; Pollock 1982). Under this formulation, the state represents the presence of conditions that allow conflicts or collisions to occur, even if events themselves are intermittent within a primary sampling season. Although some within‐season temporal variation is expected, this approach provides a practical approximation of seasonal dynamics while maintaining a sufficient number of repeated surveys to estimate detection probability.

We estimated initial occurrence (ψ_1_), emergence (known as “colonization,” *γ*_*i*_), cessation (known as “extinction,” *ε*_*i*_), and detection probability (*p*_*ij*_) using program PRESENCE via the *RPresence* package (MacKenzie and Hines 2024). Colonization (*γ*_*i*_) represents the probability that a sampling unit experiences at least one event in a primary sampling season given no event in the previous season, and extinction (*ε*_*i*_) represents the probability that events cease between primary sampling seasons. These parameters define a first‐order Markov process in which the probability of events in a given season depends on the state in the previous season, reflecting temporal dependence arising from persistent landscape structure, repeated use of space by panthers, and consistent patterns of human activity. Detection probability (*p*_*ij*_) represents the probability that a conflict or collision is detected, reported, and verified through the respective standardized monitoring system, conditional on occurrence. Imperfect detection arises because not all events are observed or reported, and because reporting effort and visibility may vary across space and time. Thus, detection probability accounts for both observation and reporting processes, rather than representing direct observation of all events.

Model parameters were allowed to change by primary sampling season (*i*), and to include singular and additive effects of selected covariates. Conflict and collision data were analyzed separately.

### 2.6 Model Selection

We followed a modeling approach guided by a priori hypotheses on covariate effects (Table S1): (1) we determined the best model structure for *p*_*ij*_ by allowing it to vary across seasons and be affected by selected covariates, while other model parameters were constrained to be constant. The best *p*_*ij*_ model was used in all subsequent steps; (2) we used a similar approach to determine the most appropriate model structure for ψ_1_, while allowing *γ*_*i*_ and *ε*_*i*_ to vary across seasons; (3) we tested the singular effect of selected covariates on *γ*_*i*_ and *ε*_*i*_, while keeping *p*_*ij*_ and ψ_1_ at the best structures determined in the previous steps; and (4) we tested for the additive effects of covariates with a discernible singular effect on *γ*_*i*_ and *ε*_*i*_.

Model selection was based on Akaike's Information Criterion (AIC; Burnham and Anderson 2002). Models with ΔAIC ≤ 2 were considered competitive. Covariate effects on model parameters were assessed by comparing AIC between models with and without a covariate, and by assessing whether the 95% confidence intervals for regression coefficients included zero.

### 2.7 Spatial Prediction

We created a predictive risk map of conflict and collision occurrence using model‐derived probabilities from the final primary sampling season (ψ_16_). Predictions were restricted to the spatial domain used for model fitting to avoid extrapolation beyond the inference domain and to ensure that predictions were made only within the range of conditions represented in the data.

All analyses were conducted in R version 4.3.2 (R Core Team 2023), and maps were produced using ArcGIS Pro 3.5.2 (ESRI 2025).

## 3 Results

### 3.1 Human–Panther Conflicts

A total of 277 conflicts were reported between October 2006 and July 2022 within the conflict grid and primary sampling seasons. The seasonal mean number of conflicts was 17.31 ± 2.88 (mean ± SE), ranging from two in 2020–2021 to 42 in 2016–2017. Depredations were the most frequently reported conflict (259, 93.5%), particularly involving goats, cattle, and sheep (Table S3). Direct interactions with humans included encounters (15, 5.4%) and incidents (3, 1.1%); no threats or attacks were reported.

**FIGURE 3:**
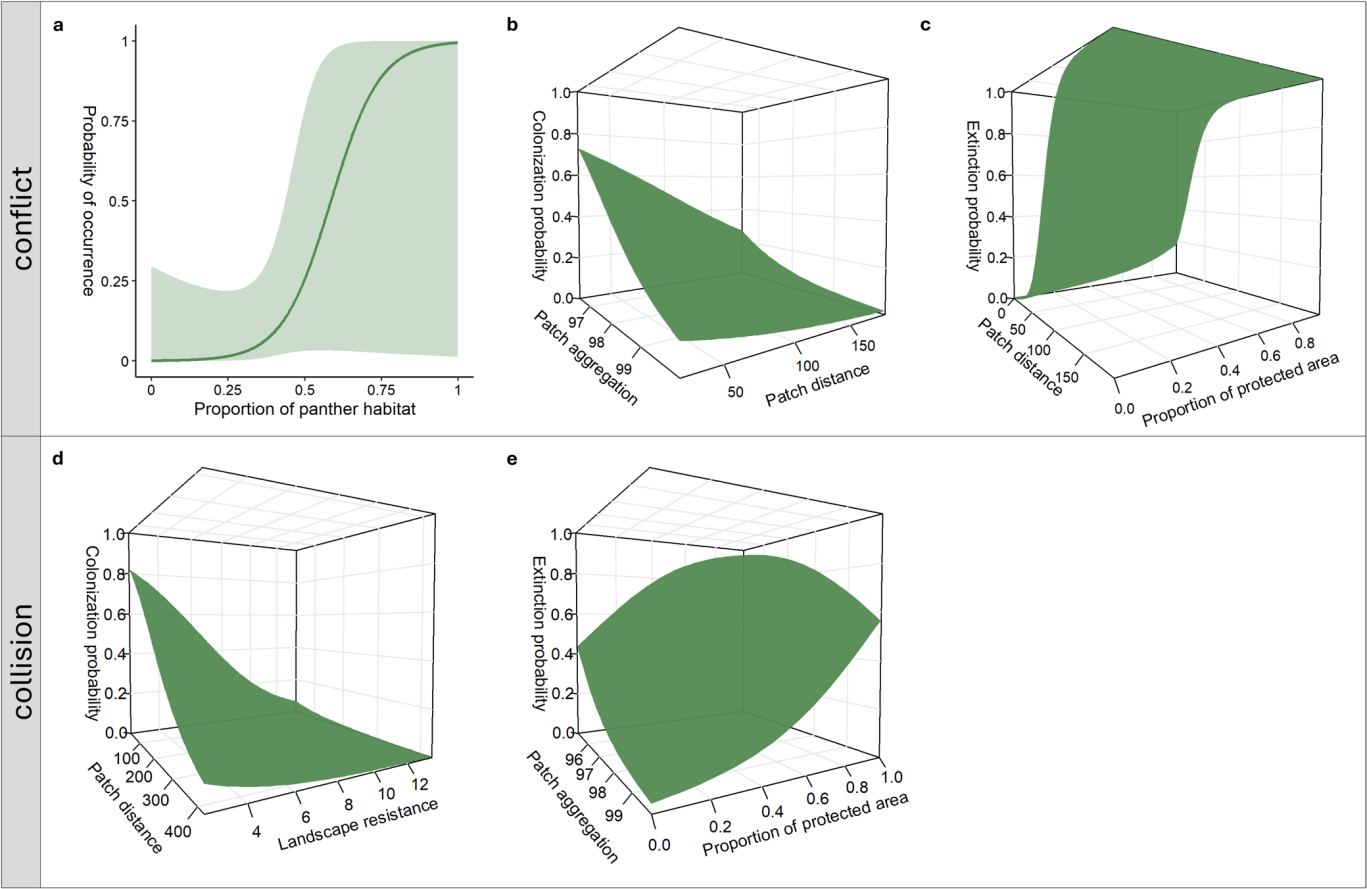
Covariate effects on occupancy parameters for human‐panther conflicts and vehicle collisions in Southwest Florida, USA. Conflicts: (a) The influence of the proportion of panther habitat on the probability of initial occurrence, (b) the influence of distance between habitat patches (m) and patch aggregation (index) on colonization probability, and (c) the influence of the proportion of protected area and distance between habitat patches (m) on extinction probability. Collisions: (d) the influence of landscape resistance (1–20 resistance score) and distance between habitat patches (m) on colonization probability; and (e) the influence of the proportion of protected area and patch aggregation (index) on extinction probability. Shaded areas indicate 95% confidence intervals.

The best‐supported conflict model was ψ_1_(proportion of panther habitat) *γᵢ*(patch aggregation + distance between patches) *εᵢ*(distance between patches + proportion of protected area) *pᵢⱼ*(proportion of protected area + season) (Table S4). The proportion of panther habitat was positively associated with initial occurrence (ψ_1_; *β* ± SE: 2.62 ± 1.66; Figure 3a). Patch aggregation (*β* ± SE: −0.66 ± 0.24) and distance between habitat patches (*β* ± SE: −0.46 ± 0.22) were negatively associated with colonization (*γᵢ*) (Figure 3b). Distance between habitat patches (*β* ± SE: 2.54 ± 0.68) and proportion of protected area (*β* ± SE: 0.84 ± 0.43) were positively associated with extinction (*εᵢ*) (Figure 3c). Detection probability (*pᵢⱼ*) was negatively associated with the proportion of protected area (*β* ± SE: −0.40 ± 0.18).

The probability of conflict occurrence slightly increased during the study period, from 0.23 in 2006–2007 to 0.29 in 2012–2013; it remained relatively constant thereafter until the end of the study (mean ± SE: 0.26 ± 0.01; Figure S1). The colonization probability did not significantly change during the study period, with a grid cell with no conflict in one season having a 15% average probability of conflict emergence in the next season (mean ± SE: 0.15 ± 0.02). Similarly, the extinction probability did not change significantly, with a grid cell that had conflict in one season having a 54% average probability of conflict cessation in the next season (mean ± SE: 0.54 ± 0.05). The detection probability varied substantially, ranging from 0.01 in 2020–2021 to 0.19 in 2016–2017 (mean ± SE: 0.10 ± 0.01; Figure S1).

### 3.2 Vehicle Collisions

A total of 239 panther deaths and injuries from collisions were reported between October 2006 and July 2022 within the road segments and primary sampling seasons. Male subadult panthers comprised the highest proportion of collisions (34.7%), with males accounting for 56.9% and females 42.3% of all collisions (Table SD5). Reported seasonal collisions ranged from four in 2007–2008 to 26 in 2015–2016, with an overall mean of 14.94 ± 1.45 (mean ± SE).

The best‐supported collision model was ψ_1_(.) *γᵢ*(landscape resistance + distance between patches) *εᵢ*(patch aggregation + proportion of protected area) *pᵢⱼ*(season) (Table S4). None of the tested covariates was associated with initial occurrence (ψ_1_). Landscape resistance (*β* ± SE: −0.95 ± 0.34) and distance between habitat patches (*β* ± SE: −0.59 ± 0.30) were negatively associated with colonization (*γᵢ*; Figure 3d). Patch aggregation (*β* ± SE: −0.72 ± 0.31) and proportion of protected area (*β* ± SE: 1.15 ± 0.36) showed opposing associations with extinction (*εᵢ*; Figure 3e). None of the tested covariates was associated with detection probability.

The probability of collision occurrence slightly increased during the early part of the study period, from 0.49 in 2006–2007 to 0.56 in 2011–2012, and remained relatively constant thereafter (mean ± SE: 0.53 ± 0.01; Figure S1). The colonization probability did not vary substantially during the study, with a road segment that had no collisions in one season having an average 29% probability of collision emergence in the next season (mean ± SE: 0.29 ± 0.02). Similarly, the extinction probability did not vary substantially, with a road segment that had collisions in one season having an average 23% probability of collision cessation in the next season (mean ± SE: 0.23 ± 0.02). Detection probability ranged from 0.01 in 2007–2008 to 0.06 in 2015–2016 (mean ± SE: 0.03 ± 0.003; Figure S1).

### 3.3 Spatial Prediction of Conflict and Collision Risk

**FIGURE 4:**
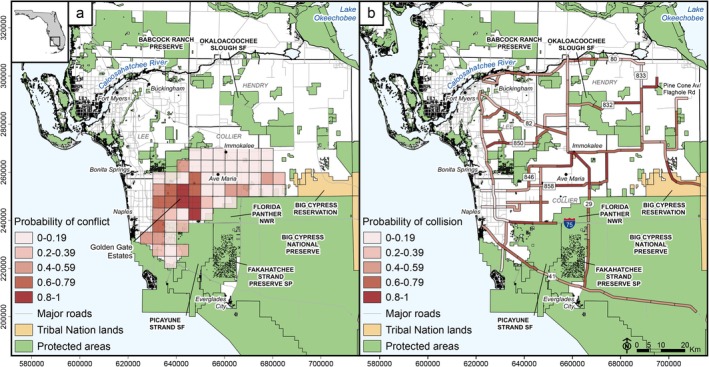
Predicted map of human‐panther conflicts and vehicle collisions in Southwest Florida, USA, derived from occupancy estimates of the last season (ψ_16_, 2021–2022) of multi‐season occupancy models: (a) probability of conflict; and (b) probability of collision. NWR, National Wildlife Refuge; SF, State Forest; SP, State Park.

Using the best‐supported models for conflicts and collisions, we generated predictive maps using derived occurrence estimates for the final primary sampling season (ψ_16_) to identify areas with high probabilities of conflict and collision occurrence across the study area (Figure 4). High probabilities of conflict occurrence (≥ 0.80) were identified in and around Golden Gate Estates in Collier County. Similarly, several road segments were predicted to have high probabilities of collision occurrence (≥ 0.80): (1) south of Immokalee and near Ave Maria (SR29, CR846, and CR858); (2) segments near the terminus of wildlife fencing along Interstate 75 near Picayune Strand State Forest; and (3) segments along CR850, CR833, CR832, and Pine Cone Avenue/Flaghole Road.

## 4 Discussion

The continued expansion of human development in Southwest Florida, together with the approximately five‐fold increase in the panther population since the 1990s, has increased the potential for conflicts with panthers and their exposure to collisions (Onorato et al. 2010, 2024; van de Kerk et al. 2019). Our results indicate that these processes are widespread within the focal landscape, with collisions occurring more frequently than conflicts (average probability of occurrence: 0.53 vs. 0.26). Both conflicts and collisions increased during the early part of the study period and subsequently remained relatively constant. Because colonization and extinction were modeled as time‐invariant in the best‐supported models, these temporal patterns should be interpreted as reflecting the balance between colonization (*γ*_*i*_) and extinction (*ε*_*i*_) dynamics rather than evidence of directional temporal change.

This temporal pattern provides insight into how anthropogenic risks can develop during carnivore population recovery. In our study, both conflicts and collisions rose alongside panther recovery, yet population size alone did not have a significant effect in our models. Instead, conflicts and collisions appear to be driven primarily by the interaction between panther movement and landscape configuration. This suggests that once populations become established within a landscape, these processes could reach dynamic equilibrium, reflecting a balance among other factors such as habitat connectivity, movement behavior, and interactions with humans (Vasudev et al. 2022).

For conflicts, landscape configuration was strongly associated with both the emergence and continued occurrence of conflicts across seasons. Landscapes characterized by high fragmentation and short distances between habitat patches were associated with higher probabilities of emerging conflicts (Figure 3b). These habitat configurations could facilitate panther movement while increasing the availability of forest edges for stalking prey, potentially increasing the likelihood of conflicts such as livestock predation (Frakes et al. 2015; Jacobs et al. 2015). In contrast, greater distances between habitat patches and higher proportions of protected area were associated with higher cessation probabilities. These patterns are consistent with the idea that fragmented or transitional landscapes may be used more intermittently, potentially by dispersing individuals, as has been suggested in other cougar populations (e.g., Suraci et al. 2025; Thompson and Jenks 2010), whereas protected areas may reduce opportunities for repeated conflicts due to lower densities of humans and domestic animals.

Our results provide limited support for population size as a primary driver of conflict, consistent with previous findings (Graham et al. 2005) and with evidence that management strategies aimed at reducing predator abundance often fail to reduce conflicts (Lennox et al. 2018; Treves and Karanth 2003). Alternative approaches, such as improved livestock husbandry, increased wild prey abundance, or economic incentives, may prove more effective, either individually or in combination (Woodroffe et al. 2005).

Across Southwest Florida, FWC established an education and outreach program to inform the public and support strategies to more effectively protect pets and livestock, as well as a pilot Panther Compensation Program to provide financial assistance to ranchers who lose free‐ranging cattle to panthers. These programs have had a special focus in and around the Golden Gate Estates, an area documented as having the highest number of human‐panther conflicts (Rodgers and Pienaar 2017, 2018). This area was also identified by our predictive model to have the highest probability of conflicts (Figure 4a), underscoring the need for managers to continue outreach and management efforts in Golden Gate Estates and surrounding areas to help reduce the probability of conflicts and increase the prospects for continued coexistence.

Similar to conflicts, collision dynamics were strongly associated with landscape characteristics and configuration. Road segments embedded in landscapes with lower resistance to panther movement and shorter distances between habitat patches were associated with higher probabilities of emerging collisions (Figure 3d). This is consistent with the idea that panthers may be more likely to move through areas where habitat connectivity and suitability is relatively high (Criffield et al. 2018; Kautz et al. 2006; Onorato et al. 2024). Persistent collision occurrence across seasons was also associated with landscape configuration, with collisions more likely to persist in areas characterized by greater patch aggregation, especially outside of protected lands.

Contrary to our expectations, traffic volume, road and shoulder width, and road fencing were not strongly associated with collision occurrence. Collisions were reported across a wide range of road types, suggesting that landscape context and panther movement patterns may be more important determinants of collision risk than road‐specific attributes. The proportion of fencing associated with wildlife crossings represented a relatively small proportion of the total area for most road segments, which may have limited our ability to detect its localized effectiveness. Nevertheless, our results identified several road segments with high probabilities of collision occurrence, particularly in areas critical for connectivity between the core panther population on public lands in the south and suitable habitat on private lands to the north (Swanson et al. 2008; Thatcher et al. 2009). Maintaining connectivity across this region is essential for facilitating northward expansion and meeting recovery objectives for the species (USFWS 2008). To ensure panther habitat connectivity, future collision mitigation strategies should consider habitat configuration and resistance to panther movement along high‐risk road segments to assist with strategically constructing additional wildlife crossings with associated fencing, which are widely used and effective at reducing road‐related mammal mortality (Lotz et al. 1997; Rytwinski et al. 2016).

The application of a dynamic occupancy modeling framework provided insights into the spatiotemporal dynamics of conflicts and collisions while accounting for imperfect detection. Detection probabilities were consistently low for both processes (Figure S1), indicating that reported events likely underestimate the total number of conflicts and collisions (Goswami et al. 2015; Moore et al. 2018). In addition, detection probability for conflicts decreased with increasing proportion of protected area, suggesting that interactions occurring near or within protected lands may be underreported.

Taken together, these findings indicate that conflicts and collisions are both associated with how landscape configuration shapes movement and spatial overlap, but they differ in the contexts in which risk is realized: conflicts occurring where human activities intersect with suitable habitat patches, and collisions occurring where movement pathways intersect with transportation infrastructure. This distinction helps explain why some areas may experience high conflict risk but relatively low collision risk, and vice versa, despite being embedded within the same broader landscape. This underscores the need for risk‐specific management approaches that account for habitat configuration, connectivity, and movement pathways. As human development continues to expand across current and potential panther range (Davis et al. 2021; Frakes et al. 2015), maintaining habitat connectivity while minimizing conflict and anthropogenic mortality will be critical for promoting dispersal, enhancing genetic diversity, and supporting long‐term population persistence (Beier 1995; Onorato et al. 2010, 2024; Schwab and Zandbergen 2011). More broadly, our results suggest that human–wildlife conflicts and collision risk are emergent outcomes arising from how recovering populations interact with heterogeneous landscapes, providing a general framework for anticipating and managing risk in large carnivore recoveries worldwide.

## Author Contributions

**Marta Prat‐Guitart:** conceptualization (lead), data curation (supporting), formal analysis (lead), funding acquisition (lead), investigation (lead), methodology (equal), visualization (lead), writing – original draft (lead). **David P. Onorato:** data curation (lead), writing – original draft (supporting). **James E. Hines:** methodology (equal), validation (supporting), writing – original draft (supporting). **Madan K. Oli:** conceptualization (equal), methodology (equal), supervision (lead), writing – original draft (supporting).

## Funding

This work was supported by “la Caixa” Foundation (LCF/BQ/AN15/10380035).

## Conflicts of Interest

The authors declare no conflicts of interest.

## Supporting information


**Table S1:** Covariates and hypothesized influence of site and seasonal covariates on dynamic occupancy parameters used to assess the drivers of human–panther conflicts and vehicle collisions in Southwest Florida, USA. Covariates were tested for both conflicts and collisions analysis unless noted otherwise. Parameters include probability of initial occurrence (ψ_1_), emergence (colonization, *γ*_*i*_), cessation (extinction, *ε*_*i*_), and detection probability (*p*_*ij*_).
**Table S2:** Land cover classes from the Cooperative Land Cover Layer (FWC 2016), reclassified for the analysis of human–panther conflicts and vehicle collisions in Southwest Florida, USA. The habitat category was used to define land covers favorable to the Florida panther (1) and unfavorable (0). The resistance category was used to define resistance to panther movement (1–20), with lower values representing lower movement resistance. Land cover classes not found within the study area were not included.
**Table S3:** Reported panther depredations by species within the conflict grid in Southwest Florida, USA, between October 2006–July 2022, excluding the months of August and September.
**Table S4:** Model selection results for the 5 top multi‐season occupancy models used to assess covariate effects on initial occurrence (ψ_1_), emergence (colonization, *γ*_*i*_), cessation (extinction, *ε*_*i*_), and detection probability (*p*_*ij*_) of (a) human–panther conflicts, and (b) vehicle collisions, in Southwest Florida, USA. K is the number of estimated parameters, AIC is the Akaike Information criterion, ΔAIC is the difference in AIC statistics between the most parsimonious model and a selected model, and *wi* is the model weight.
**Table S5:** Reported Florida panther vehicle collisions within the collisions grid in Southwest Florida, USA, between October 2006–July 2022, excluding the months of August and September.
**Figure S1:** Probability of occurrence (a) and detection probability (b) across primary sampling seasons (*i*) for human–panther conflicts (orange) and vehicle collisions (green) in Southwest Florida, USA.

## Data Availability

The data that support the findings of this study are openly available in Figshare: https://doi.org/10.6084/m9.figshare.32527017. Florida panther mortality data were derived from sources openly available in the public domain: https://geodata.myfwc.com/.
