# Supplementary material for: Conflicts and Collisions With an Endangered Carnivore: Landscape Drivers and Spatial Risk Pattern
Source: Ecol Evol. 2026 Jul 3;16(7):e73931. doi: 10.1002/ece3.73931 (PMC13329263; doi:10.1002/ece3.73931)
Supplement: Supplementary file 1 — Table S1: Covariates and hypothesized influence of site and seasonal covariates on dynamic occupancy parameters used to assess the drivers of human–panther conflicts and vehicle collisions in Southwest Florida, USA. Covariates were tested for both conflicts and collisions analysis unless noted otherwise. Parameters include probability of initial occurrence (ψ1), emergence (colonization, γ i ), cessation (extinction, ε i ), and detection probability (p ij ). Table S2: Land cover classes from the Cooperative Land Cover Layer (FWC 2016), reclassified for the analysis of human–panther conflicts and vehicle collisions in Southwest Florida, USA. The habitat category was used to define land covers favorable to the Florida panther (1) and unfavorable (0). The resistance category was used to define resistance to panther movement (1–20), with lower values representing lower movement resistance. Land cover classes not found within the study area were not included. Table S3: Reported panther depredations by species within the conflict grid in Southwest Florida, USA, between October 2006–July 2022, excluding the months of August and September. Table S4: Model selection results for the 5 top multi‐season occupancy models used to assess covariate effects on initial occurrence (ψ1), emergence (colonization, γ i ), cessation (extinction, ε i ), and detection probability (p ij ) of (a) human–panther conflicts, and (b) vehicle collisions, in Southwest Florida, USA. K is the number of estimated parameters, AIC is the Akaike Information criterion, ΔAIC is the difference in AIC statistics between the most parsimonious model and a selected model, and wi is the model weight. Table S5: Reported Florida panther vehicle collisions within the collisions grid in Southwest Florida, USA, between October 2006–July 2022, excluding the months of August and September. Figure S1: Probability of occurrence (a) and detection probability (b) across primary sampling seasons (i) for human–panther conflicts [file ECE3-16-e73931-s001.pdf]

SUPPLEMENTARY MATERIAL

Table S1. Covariates and hypothesized influence of site and seasonal covariates on dynamic occupancy parameters used to assess the drivers of human-panther conflicts and vehicle collisions in Southwest Florida, USA. Covariates were tested for both conflicts and collisions analysis unless noted otherwise. Parameters include probability of initial occurrence (ψ_1), emergence (colonization, γ_i), cessation (extinction, ε_i), and detection probability (p_{ij}).

Covariate	Hypothesis	Parameter
Panther population estimate	Conflicts and collisions occurrence increase with the number of panthers in the landscape	γ_i, ε_i
Proportion of panther habitat	Favorable habitats for panthers increase the occurrence of conflicts and collisions	ψ_1, γ_i, ε_i, p_{ij}
Proportion of protected area	Occurrence and detection are reduced in areas with more protected area	ψ_1, γ_i, ε_i, p_{ij}*
Landscape resistance	Increased landscape resistance to panther movement reduces the occurrence of conflicts and collisions	ψ_1, γ_i, ε_i
Least cost distance to protected area*	As distance from protected areas increases, the occurrence of conflicts is reduced	ψ_1, γ_i, ε_i
Patch aggregation	More panther movement is likely to occur in areas with higher patch aggregation, increasing the occurrence of conflicts and collisions	ψ_1, γ_i, ε_i

Distance between patches	More panther movement is likely to occur between favorable habitat at shorter distances, increasing conflict and collision occurrence	$\psi_1, \gamma_i, \varepsilon_i$
Maximum temperature	Higher temperatures encourage panther movement at nighttime, which increase the occurrence of conflicts and collisions	γ_i, ε_i
Total precipitation	Increased precipitation limits panther movement to upland areas, increasing the occurrence of conflicts and collisions	γ_i, ε_i
Proportion of fencing around wildlife crossings[a]	Wildlife crossings with higher proportion of fencing limit panther road crossings and reduce the occurrence of collisions	$\psi_1, \gamma_i, \varepsilon_i$
Road width[a]	Increased road width increases time and distance for panthers to cross the road, increasing the occurrence of collisions	$\psi_1, \gamma_i, \varepsilon_i$
Road shoulder width[a]	The visual range around a road is wider with increased shoulder width, increasing the ability of drivers to react, reducing the occurrence of collisions	$\psi_1, \gamma_i, \varepsilon_i$
Maximum speed limit[a]	Higher speed limits reduce driver response time, increasing the occurrence of collisions	$\psi_1, \gamma_i, \varepsilon_i, p_{ij}$
Human density	Areas with lower human densities, such as rural or exurban development, provide continuous green space, increasing the occurrence of conflicts. Similarly, higher	$\gamma_i, \varepsilon_i, p_{ij}$

	human densities increase human movement, increasing the occurrence of collisions	
Livestock heads*	Larger livestock production increases the number of calves on pastures, increasing the occurrence of conflicts	γ_{i}, ε_{i}
Traffic volume[a]	A larger number of cars driving on the road increases the occurrence and detection of collisions	γ_{i}, ε_{i}, p_{ij}

* Covariate included only in conflict models.

[a] Covariate included only in collision models.

Table S2. Land cover classes from the Cooperative Land Cover Layer (FWC, 2016), reclassified for the analysis of human-panther conflicts and vehicle collisions in Southwest Florida, USA. The habitat category was used to define land covers favorable to the Florida panther (1) and unfavorable (0). The resistance category was used to define resistance to panther movement (1-20), with lower values representing lower movement resistance. Land cover classes not found within the study area were not included.

CLC value	Land Cover Class	Habitat	Resistance
1110	Upland Hardwood Forest	1	1
1112	Mixed Hardwoods	1	1
1120	Mesic Hammock	1	1
1122	Prairie Mesic Hammock	1	4
1123	Live Oak	1	4
1124	Pine - Mesic Oak	1	3
1125	Cabbage Palm	1	2
1130	Rockland Hammock	1	2
1150	Xeric Hammock	1	1
1210	Scrub	0	6
1213	Sand Pine Scrub	1	2
1214	Coastal Scrub	0	10
1300	Pine Flatwoods and Dry Prairie	1	1
1311	Mesic Flatwoods	1	1
1312	Scrubby Flatwoods	1	1
1320	Pine Rockland	1	2
1330	Dry Prairie	1	4
1340	Palmetto Prairie	1	4
1400	Mixed Hardwood-Coniferous	1	1
1410	Successional Hardwood Forest	1	4
1500	Shrub and Brushland	1	6
1510	Other Shrubs and Brush	0	6
1600	Coastal Uplands	0	10
1610	Beach Dune	0	10
1620	Coastal Berm	0	10
1630	Coastal Grassland	0	10
1640	Coastal Strand	0	10
1650	Maritime Hammock	0	10
1660	Shell Mound	0	10

1670	Sand Beach (Dry)	0	10
1750	Bare Soil	0	3
1800	Cultural - Terrestrial	0	10
1810	Mowed Grass	0	10
1811	Vegetative Berm	0	4
1812	Highway Rights of Way	0	20
1821	Low Intensity Urban	0	10
1822	High Intensity Urban	0	20
1831	Rural Open	0	7
1832	Rural Structures	0	7
1840	Transportation	0	20
1850	Communication	0	20
1860	Utilities	0	20
1870	Extractive	0	20
1871	Strip Mines	0	20
1872	Sand & Gravel Pits	0	20
1873	Rock Quarries	0	20
1874	Oil & Gas Fields	0	10
1875	Reclaimed Lands	0	20
1877	Spoil Area	0	20
1880	Bare Soil/Clear Cut	0	7
2100	Freshwater Non-Forested Wetlands	0	2
2110	Prairies and Bogs	0	4
2111	Wet Prairie	0	7
2112	Mixed Scrub-Shrub Wetland	1	2
2113	Marl Prairie	0	6
2120	Marshes	0	6
2121	Isolated Freshwater Marsh	0	2
2122	Coastal Interdunal Swale	0	10
2123	Floodplain Marsh	0	2
2124	Slough Marsh	0	2
2125	Glades Marsh	0	4
2131	Sawgrass	0	2
2140	Floating/Emergent Aquatic Vegetation	0	10
2141	Slough	0	10
2150	Submergent Aquatic Vegetation	0	10
2200	Freshwater Forested Wetlands	1	1
2210	Cypress/Tupelo(incl Cy/Tu mixed)	1	2
2211	Cypress	1	2
2212	Tupelo	1	2

2213	Isolated Freshwater Swamp	1	2
2214	Strand Swamp	0	10
2215	Floodplain Swamp	0	10
2220	Other Coniferous Wetlands	1	2
2221	Wet Flatwoods	1	2
2230	Other Hardwood Wetlands	1	2
2231	Baygall	0	2
2232	Hydric Hammock	1	2
2233	Mixed Wetland Hardwoods	1	2
2240	Other Wetland Forested Mixed	1	2
2241	Cypress/Hardwood Swamps	1	2
2242	Cypress/Pine/Cabbage Palm	1	2
2300	Non-vegetated Wetland	0	6
2400	Cultural - Palustrine	0	15
2410	Impounded Marsh	0	6
2450	Wet Coniferous Plantation	0	7
3000	Lacustrine	0	15
3100	Natural Lakes and Ponds	0	15
3110	Limnetic	0	15
3111	Clastic Upland Lake	0	15
3113	Flatwoods/Prairie/Marsh Lake	0	15
3114	River Floodplain Lake/Swamp Lake	0	15
3120	Littoral	0	15
3200	Cultural - Lacustrine	0	15
3210	Artificial/Farm Pond	0	15
3211	Aquacultural Ponds	0	15
3220	Artificial Impoundment/Reservoir	0	15
3230	Quarry Pond	0	15
3240	Sewage Treatment Pond	0	20
3250	Stormwater Treatment Areas	0	20
3260	Industrial Cooling Pond	0	20
4000	Riverine	0	15
4100	Natural Rivers and Streams	0	15
4120	Blackwater Stream	0	15
4160	Tidally-influenced Stream	0	15
4200	Cultural - Riverine	0	15
4210	Canal	0	15
4220	Ditch/Artificial Intermittent Stream	0	15
5000	Estuarine	0	15
5200	Intertidal	0	15

5220	Tidal Flat	0	10
5240	Salt Marsh	0	10
5250	Mangrove Swamp	0	10
5252	Scrub Mangrove	0	10
5300	Cultural - Estuarine	0	15
5310	Estuarine Ditch/Channel	0	15
6000	Marine	0	15
7000	Exotic Plants	0	8
7100	Australian Pine	0	8
7200	Melaleuca	0	8
7300	Brazilian Pepper	0	8
7400	Exotic Wetland Hardwoods	0	8
9100	Unconsolidated Substrate	0	8
18211	Urban Open Land	0	10
18212	Residential, Low Density	0	10
18213	Grass	0	10
18214	Trees	0	10
18221	Residential, Med. Density - 2-5 Dwelling Units/AC	0	15
18222	Residential, High Density > 5 Dwelling Units/AC	0	20
18223	Commercial and Services	0	20
18224	Industrial	0	20
18225	Institutional	0	20
18311	Rural Open Forested	0	7
18312	Rural Open Pine	0	7
18332	Orchards/Groves	0	7
18333	Tree Plantations	0	7
18334	Vineyard and Nurseries	0	7
18335	Other Agriculture	0	7
21121	Shrub Bog	0	6
21211	Depression Marsh	0	6
21212	Basin Marsh	0	6
22131	Dome Swamp	0	6
22132	Basin Swamp	0	6
22211	Hydric Pine Flatwoods	1	2
22212	Hydric Pine Savanna	1	2
22311	Bay Swamp	0	6
22312	South Florida Bayhead	0	6
22321	Coastal Hydric Hammock	0	10
22322	Prairie Hydric Hammock	1	4
22323	Cabbage Palm Hammock	1	2

52111	Keys Tidal Rock Barren	0	15
182111	Urban Open Forested	0	10
182112	Urban Open Pine	0	10
182131	Parks and Zoos	0	20
182132	Golf courses	0	7
182133	Ballfields	0	20
182134	Cemeteries	0	15
182135	Community rec. facilities	0	20
183111	Oak - Cabbage Palm Forests	1	2
183311	Row Crops	0	7
183312	Field Crops	0	7
183313	Improved Pasture	0	7
183314	Unimproved/Woodland Pasture	0	7
183321	Citrus	0	7
183322	Fruit Orchards	0	7
183323	Pecan	0	7
183324	Fallow Orchards	0	7
183331	Hardwood Plantations	1	7
183332	Coniferous Plantations	1	7
183341	Tree Nurseries	0	7
183342	Sod Farms	0	7
183343	Ornamentals	0	7
183351	Feeding Operations	0	7
183352	Specialty Farms	0	7
222112	Cabbage Palm Flatwoods	1	2
1833121	Sugarcane	0	7
1833151	Fallow Cropland	0	7

Table S3. Reported panther depredations by species within the conflict grid in Southwest Florida, USA, between October 2006 - July 2022, excluding the months of August and September.

**Species**	**Number of times included in a report***	**Injured and fatal head counts**
Alpaca	1	1
Axis deer	1	1
Cow/calf [a]	67	68
Cat	9	18
Chicken	3	12
Donkey	2	3
Dog	7	7
Duck	2	3
Emu	3	3
Fallow deer	1	1
Goat	133	209
Goose	4	5
Horse (adult)	2	2
Llama	1	1
Mini cattle zebu	2	2
Mini horse	11	16
Pig	6	8
Pony	1	1
Sheep	23	47
Swan	1	1
Turkey	5	6
Wallaby	1	1
Total	286	416

*Multiple species included in a single report. Total number of reports = 259

[a] This category included only two cows >1 year old

Table S4. Model selection results for the 5 top multi-season occupancy models used to assess covariate effects on initial occurrence (ψ_1), emergence (colonization, γ_i), cessation (extinction, ε_i), and detection probability (p_{ij}) of (a) human-panther conflicts, and (b) vehicle collisions, in Southwest Florida, USA. K is the number of estimated parameters, AIC is the Akaike Information criterion, ΔAIC is the difference in AIC statistics between the most parsimonious model and a selected model, and *wi* is the model weight.

**(a)**	**Model structure**	**K**	**AIC**	**ΔAIC**	**w_i**
	ψ_1(phab) γ_i(pagg+dist) ε_i(dist+pa) p_{ij}(pa+s)	25	1764.37	0	0.47
	ψ_1(phab) γ_i(pagg+dist) ε_i(dist) p_{ij}(pa+s)	24	1765.24	0.86	0.30
	ψ_1(phab) γ_i(pagg) ε_i(dist+pa) p_{ij}(pa+s)	24	1766.98	2.60	0.13
	ψ_1(phab) γ_i(pagg) ε_i(dist) p_{ij}(pa+s)	23	1767.92	3.55	0.08
	ψ_1(phab) γ_i(dist) ε_i(dist+pa) p_{ij}(pa+s)	24	1772.70	8.33	0.01
**(b)**					
	ψ_1(.) γ_i(avgcost+dist) ε_i(pagg+pa) p_{ij}(s)	23	2273.39	0	0.30
	ψ_1(.) γ_i(avgcost) ε_i(dist+pa) p_{ij}(s)	22	2275.73	2.35	0.09
	ψ_1(.) γ_i(avgcost+dist) ε_i(dist+pa) p_{ij}(s)	23	2276.38	3.00	0.07
	ψ_1(.) γ_i(dist+hd) ε_i(pagg+pa) p_{ij}(s)	23	2276.50	3.11	0.06
	ψ_1(.) γ_i(avgcost+pa) ε_i(dist+pa) p_{ij}(s)	23	2277.11	3.72	0.05

Covariate abbreviations: phab (proportion of panther habitat), pagg (patch aggregation), dist (distance between habitat patches), pa (proportion of protected area), s (change by primary sampling season, *i*), avgcost (average landscape resistance), and hd (human population density).

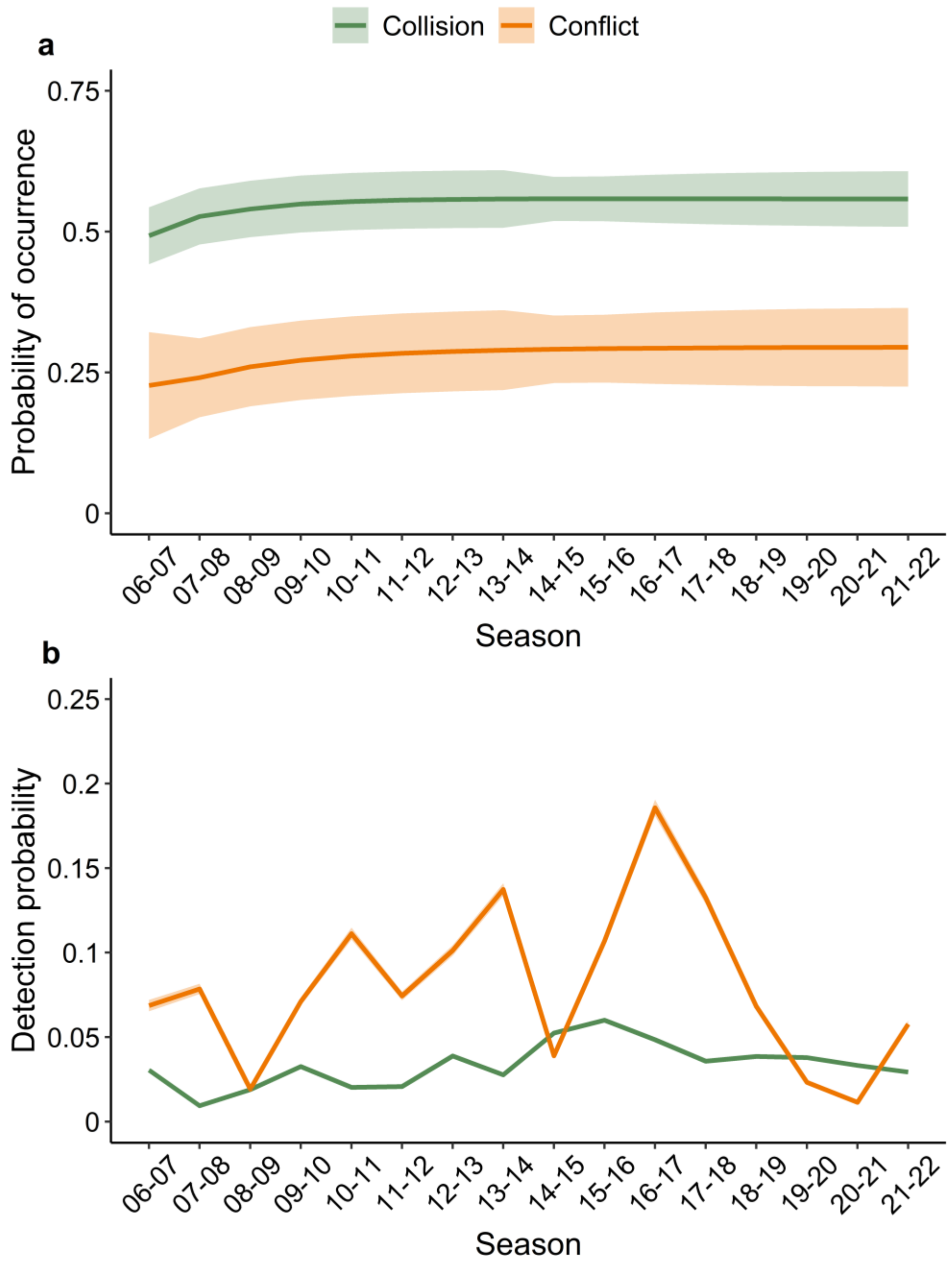

Fig. S1. Probability of occurrence (a) and detection probability (b) across primary sampling seasons (i) for human-panther conflicts (orange) and vehicle collisions (green) in Southwest Florida, USA.

Table S5. Reported Florida panther vehicle collisions within the collisions grid in Southwest Florida, USA, between October 2006 - July 2022, excluding the months of August and September.

**Age class***		**Kitten**	**Subadult**	**Adult**	**Unknown**	**Total**
Sex	Female	25	32	41	3	101
	Male	23	83	28	2	136
	Unknown				2	2

*Age classes from Hostetler *et al.* (2013): Kittens (0-1 years); Subadults (1-2.5 years for females, 1-3.5 years for males); Adults (>2.5 years for females, >3.5 years for males)

REFERENCES

FWC. (2016). Cooperative Land Cover, version 3.2. Accessed 1 October 2017. http://myfwc.com/research/gis/applications/articles/Cooperative-Land-Cover

Hostetler, J. A., Onorato, D. P., Jansen, D., & Oli, M. K. (2013). A cat's tale: the impact of genetic restoration on Florida panther population dynamics and persistence. *J. Anim. Ecol.* 82, 608–620.
